# Supplementary figures and images for: The Transcription of Flight Energy Metabolism Enzymes Declined with Aging While Enzyme Activity Increased in the Long-Distance Migratory Moth, Spodoptera frugiperda
Source: Insects. 2022 Oct 16;13(10):936. doi: 10.3390/insects13100936 (PMC9604208; doi:10.3390/insects13100936)

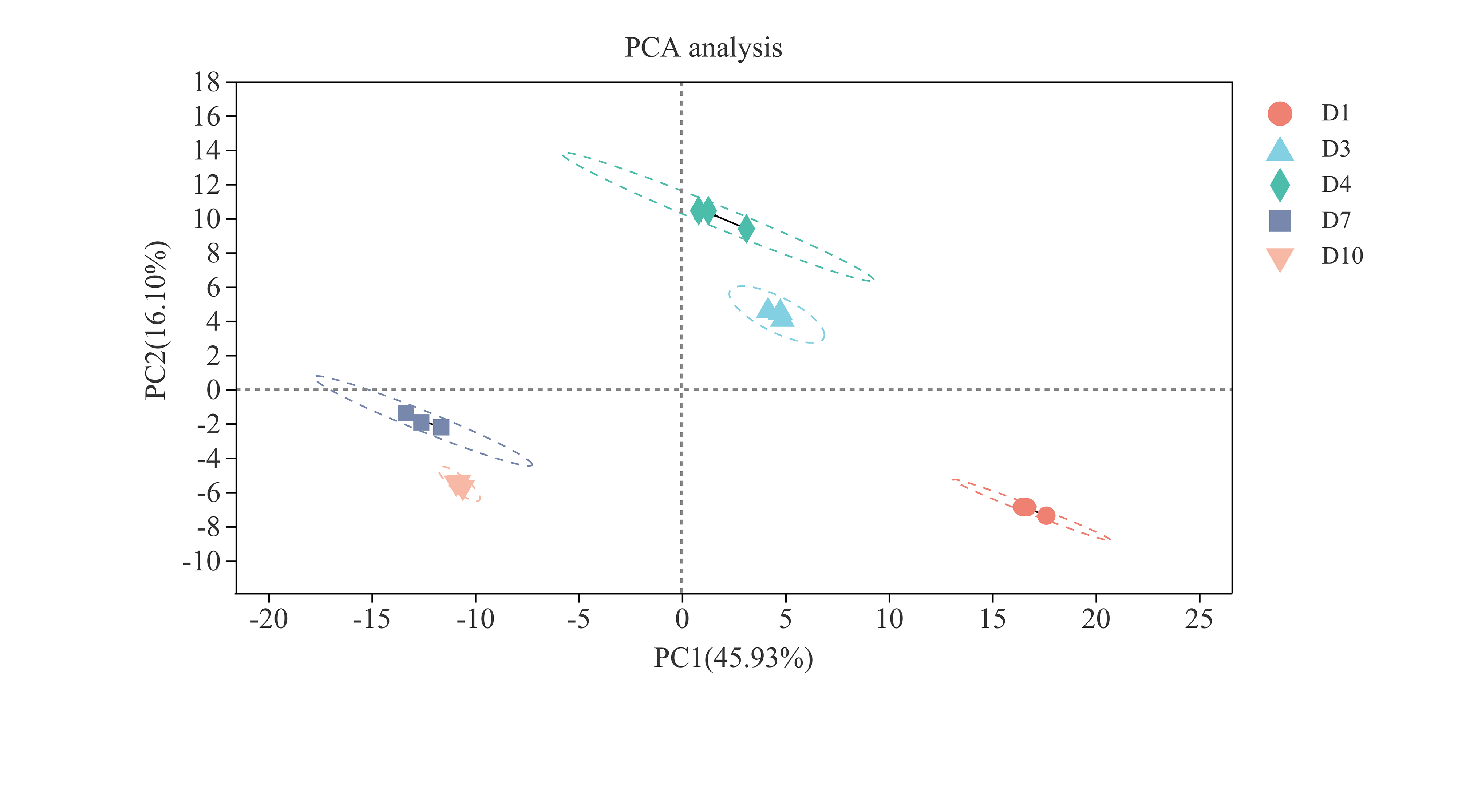

Supplement: Supplementary file 1 [file insects-13-00936-s001.zip › Fig S1.tif]

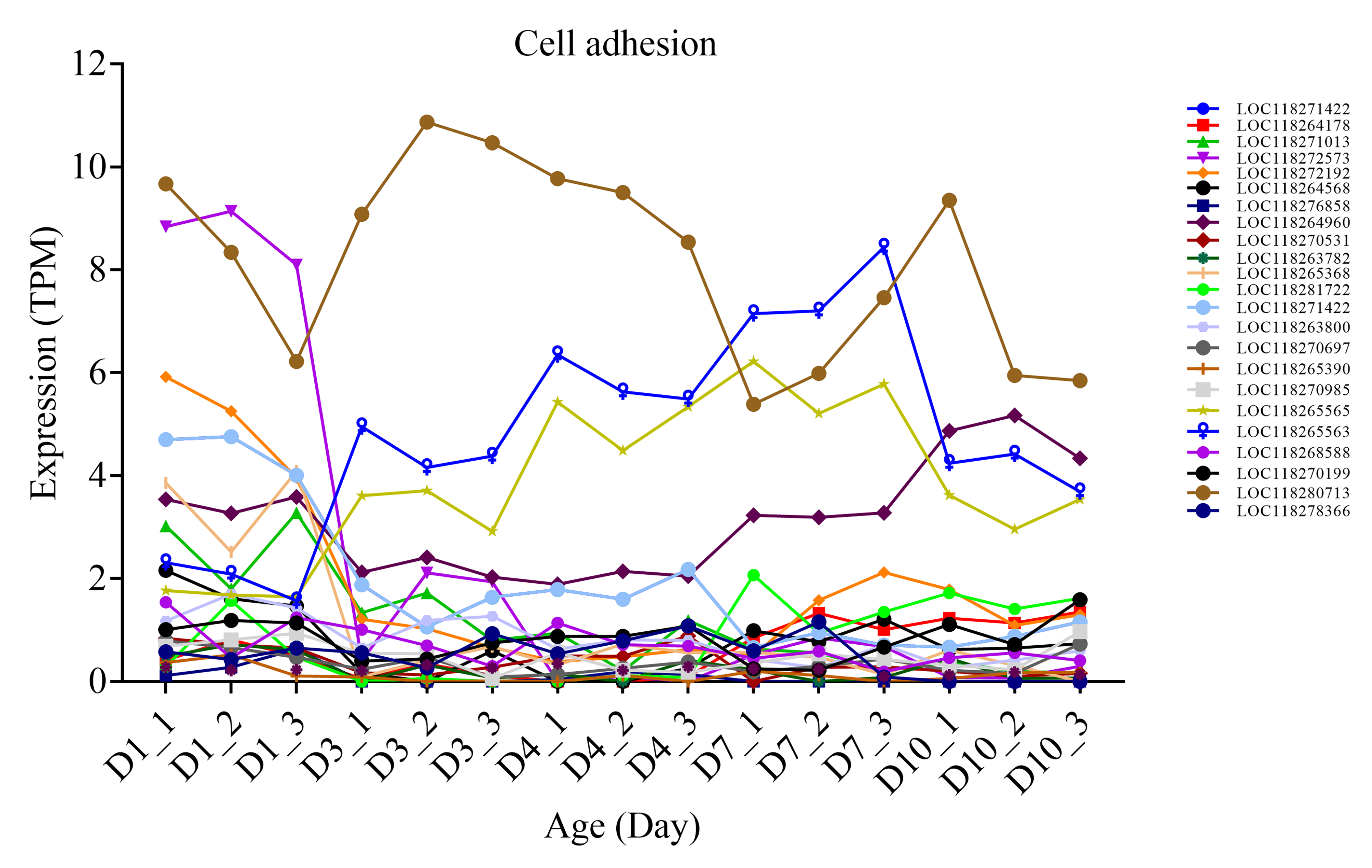

Supplement: Supplementary file 1 [file insects-13-00936-s001.zip › Fig S10.tif]

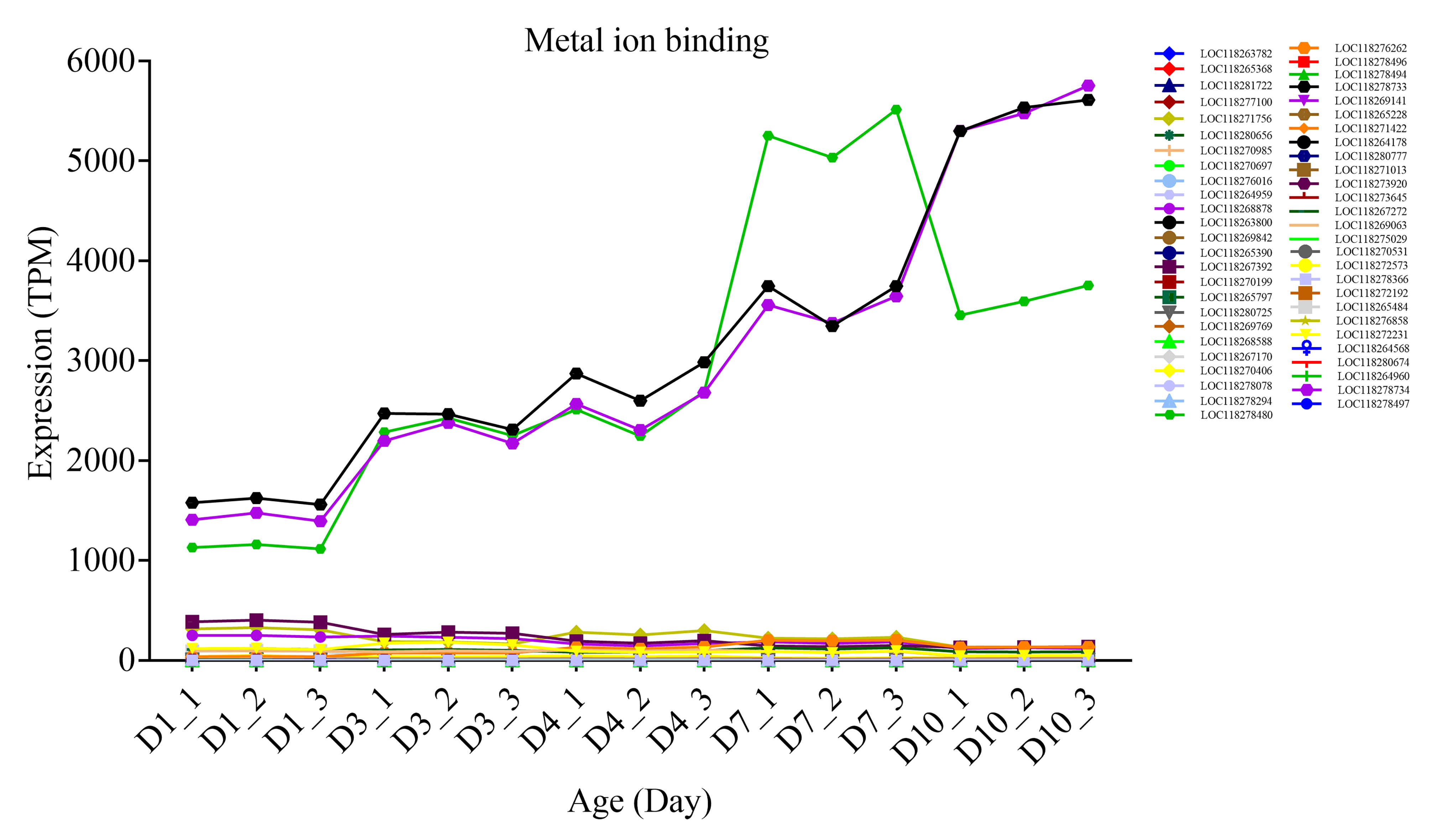

Supplement: Supplementary file 1 [file insects-13-00936-s001.zip › Fig S11.tif]

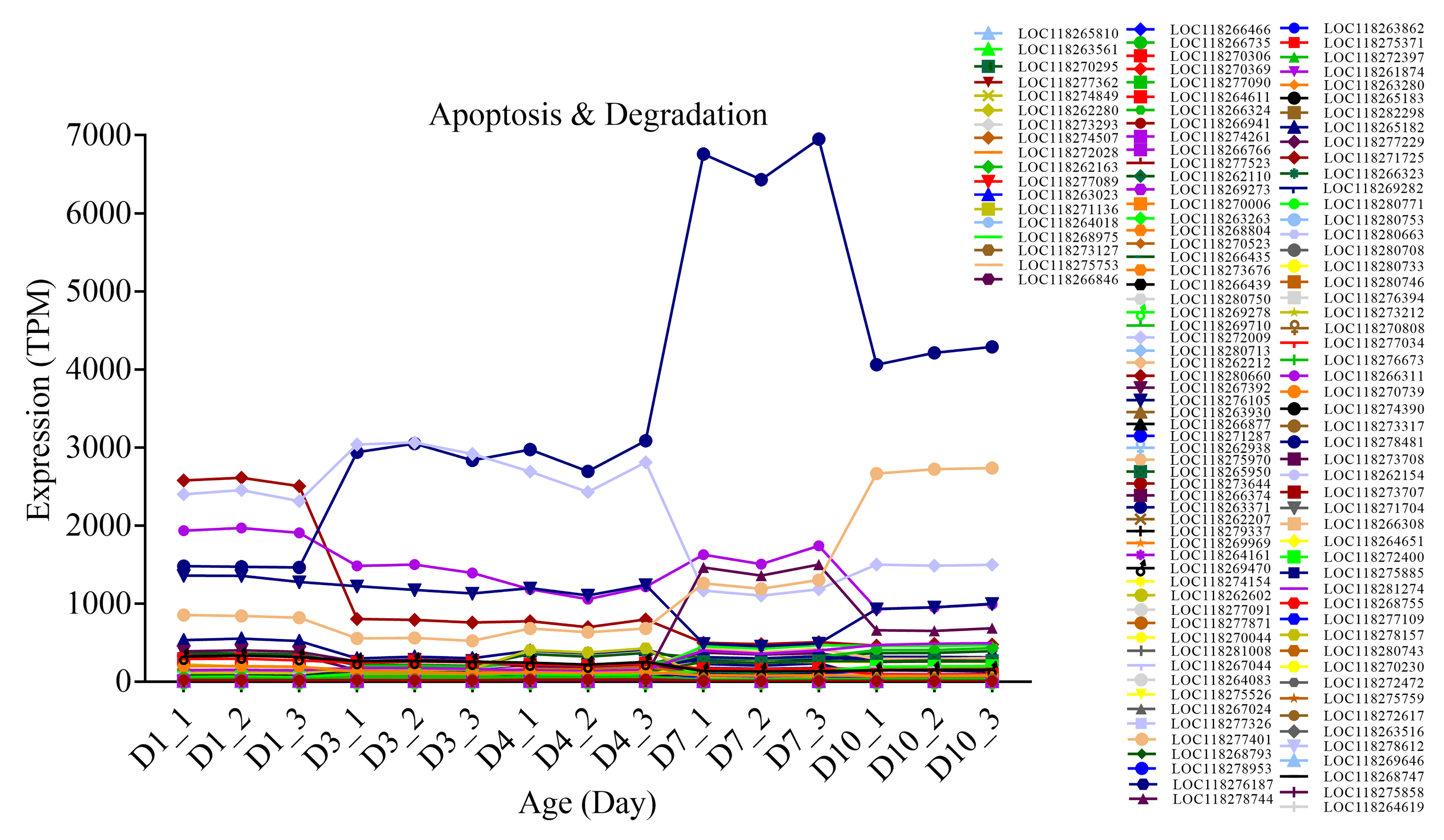

Supplement: Supplementary file 1 [file insects-13-00936-s001.zip › Fig S12.tif]

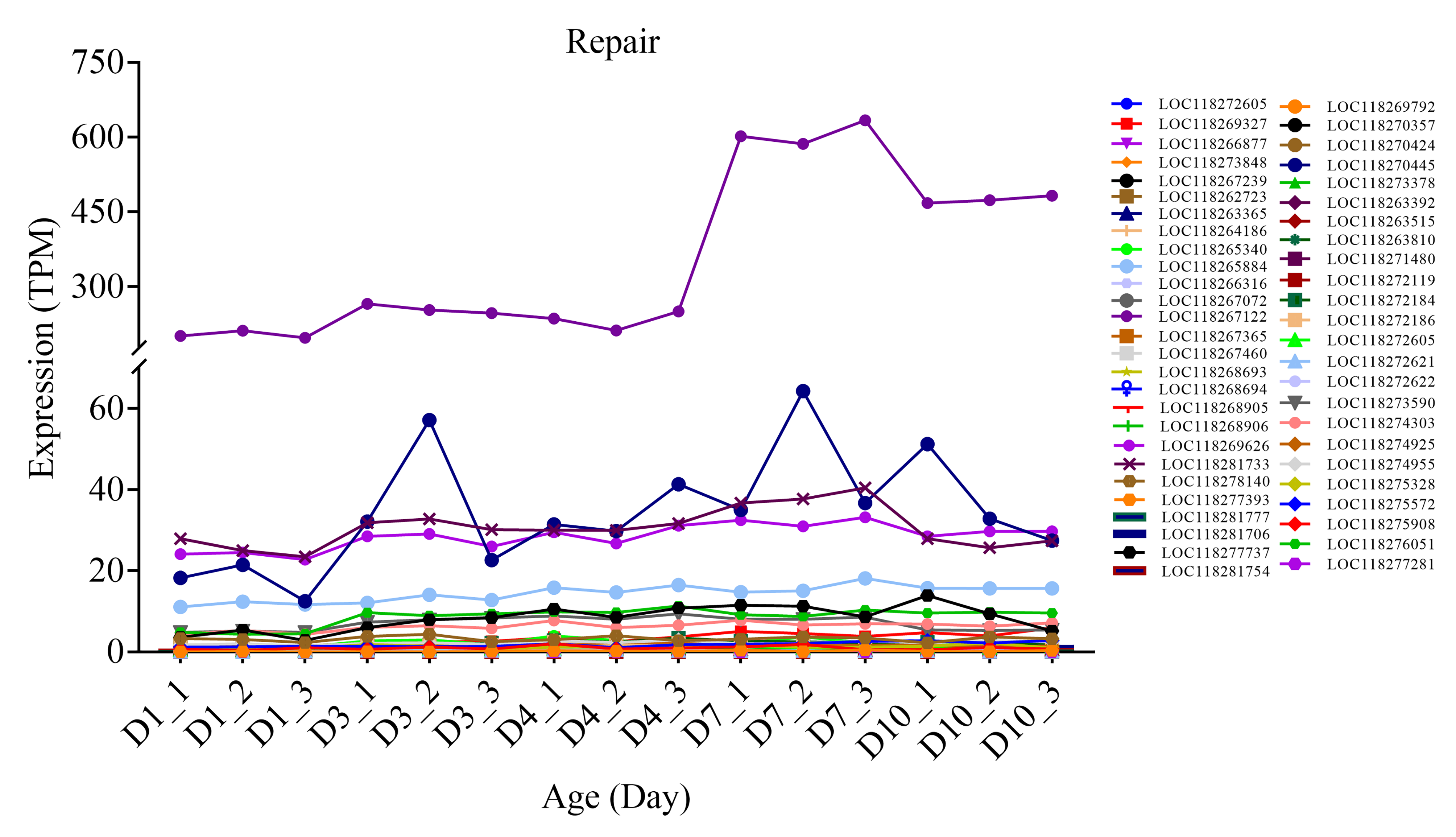

Supplement: Supplementary file 1 [file insects-13-00936-s001.zip › Fig S13.tif]

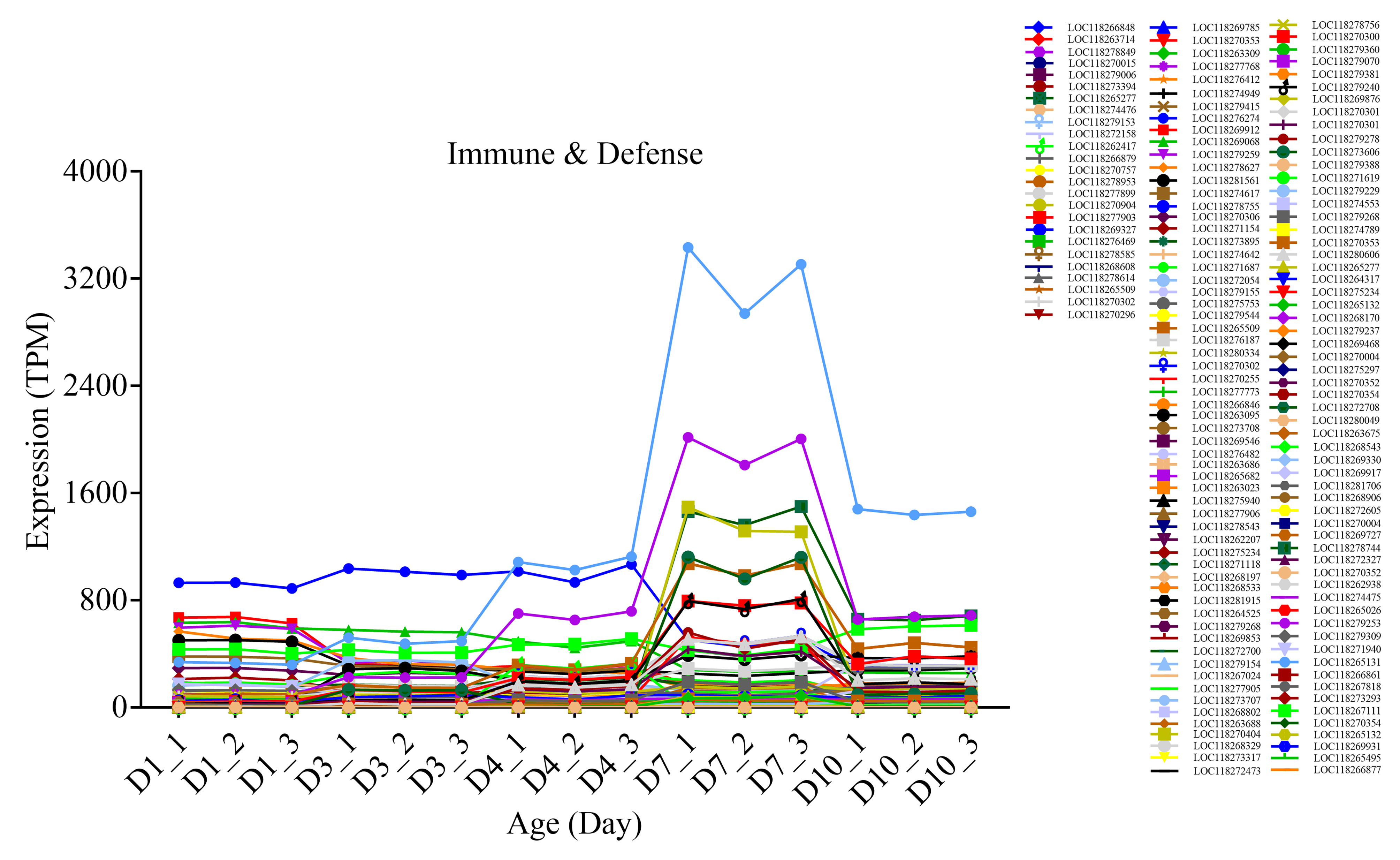

Supplement: Supplementary file 1 [file insects-13-00936-s001.zip › Fig S14.tif]

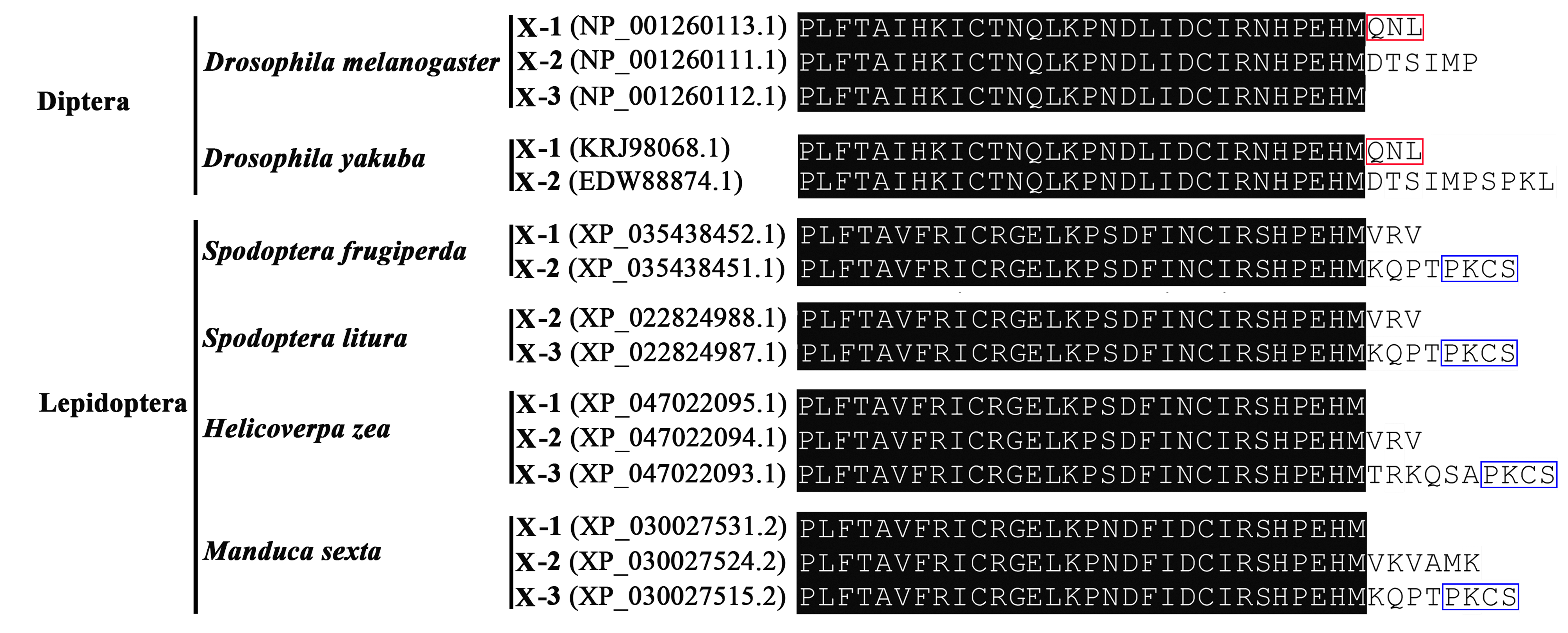

Supplement: Supplementary file 1 [file insects-13-00936-s001.zip › Fig S15.tif]

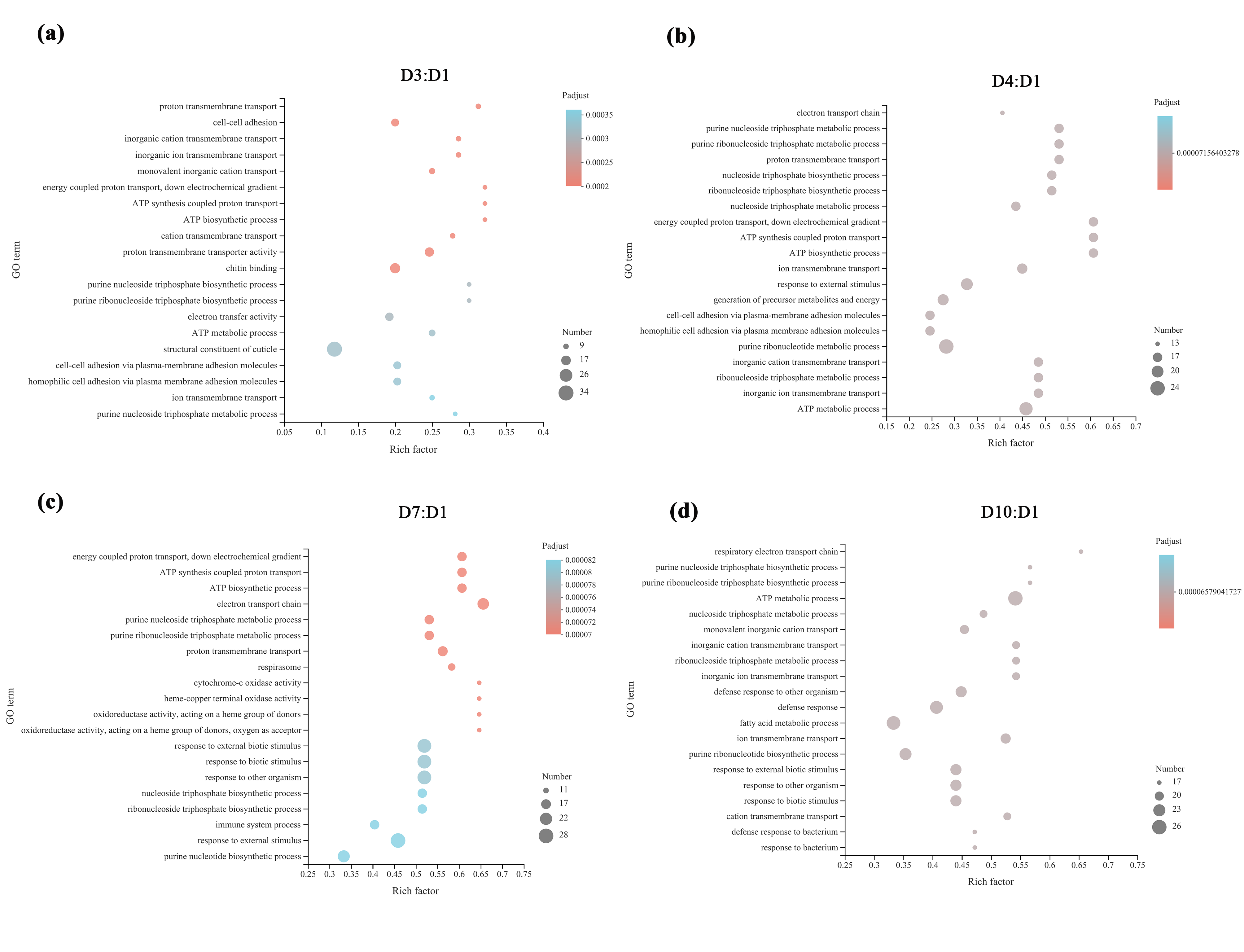

Supplement: Supplementary file 1 [file insects-13-00936-s001.zip › Fig S2.tif]

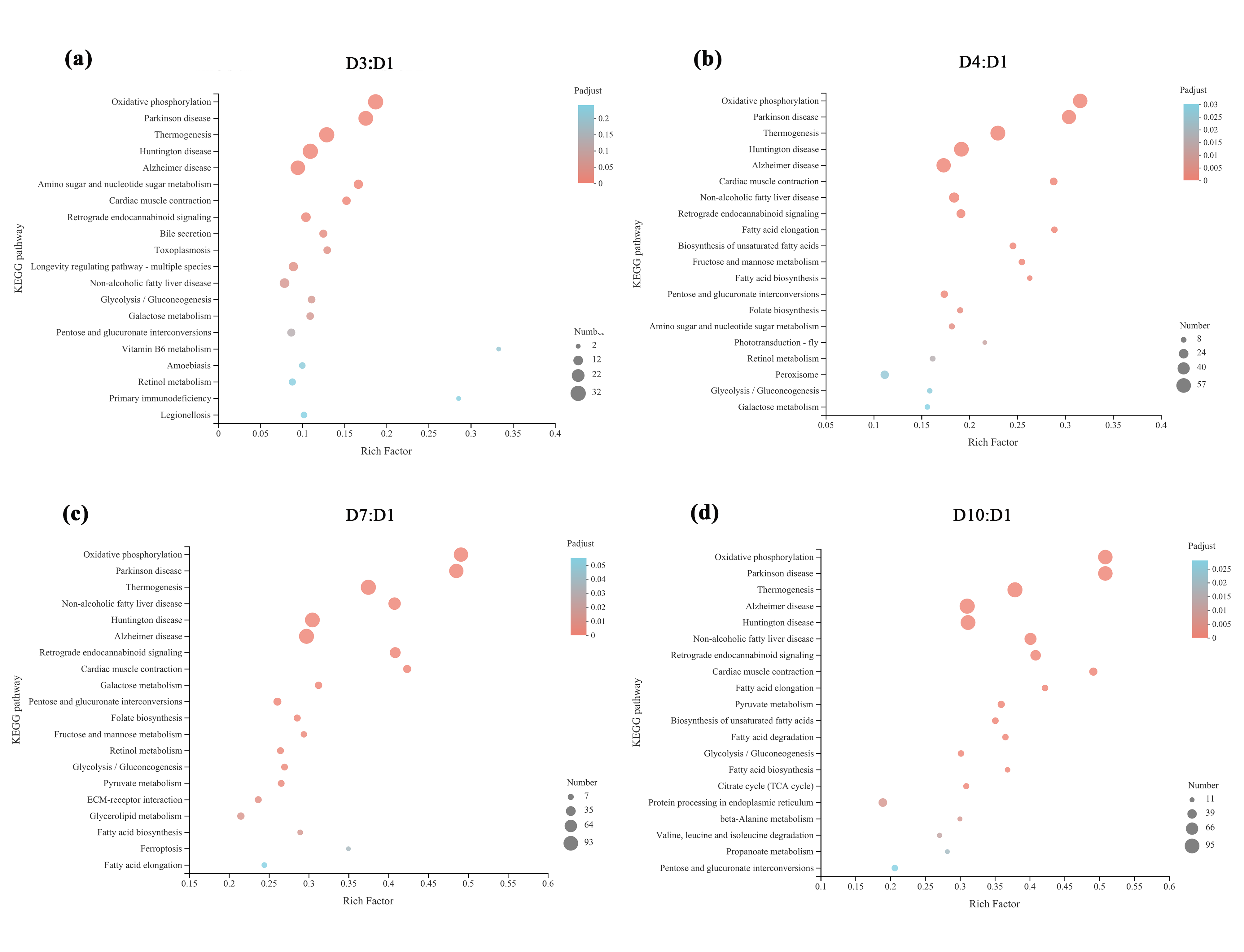

Supplement: Supplementary file 1 [file insects-13-00936-s001.zip › Fig S3.tif]

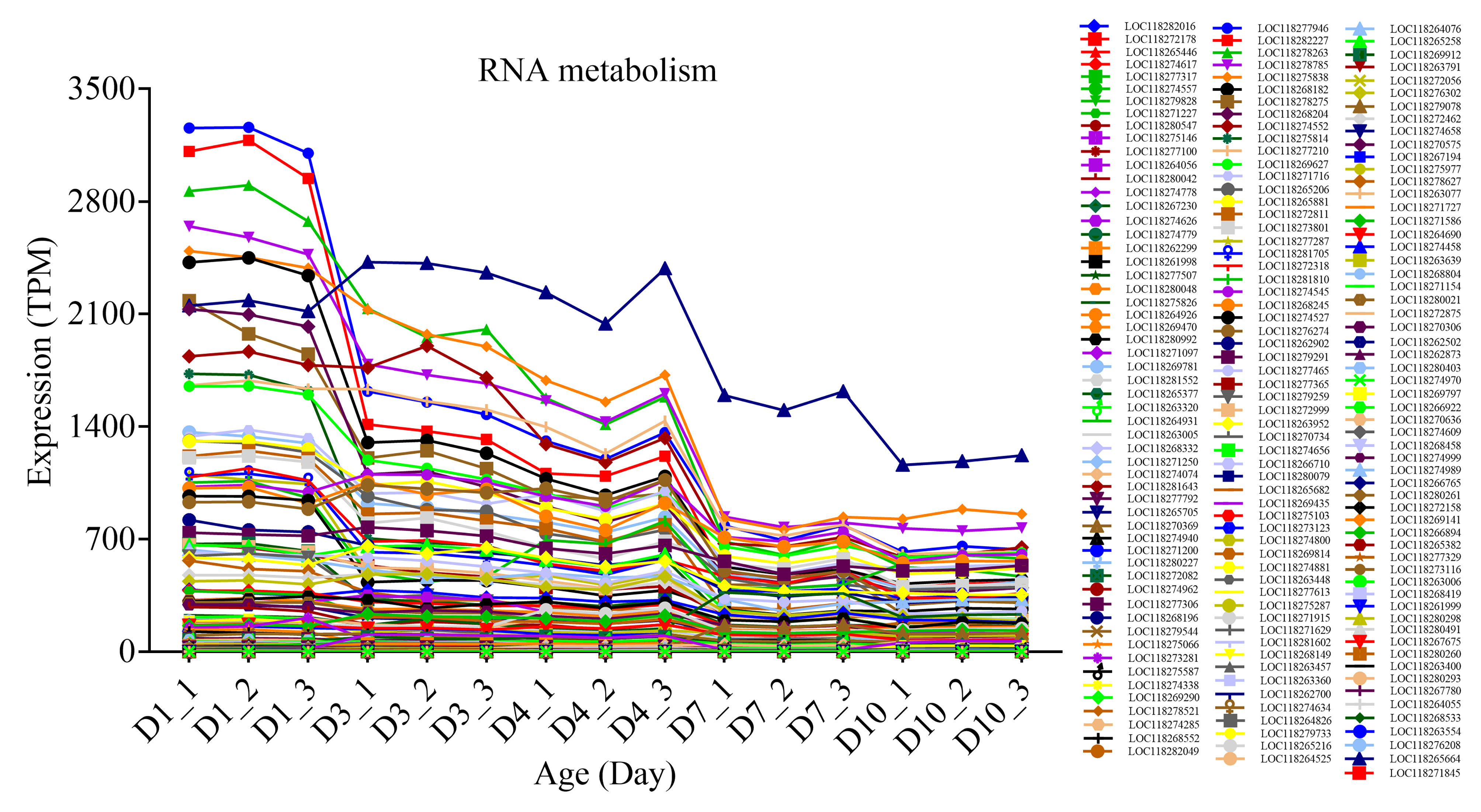

Supplement: Supplementary file 1 [file insects-13-00936-s001.zip › Fig S4.tif]

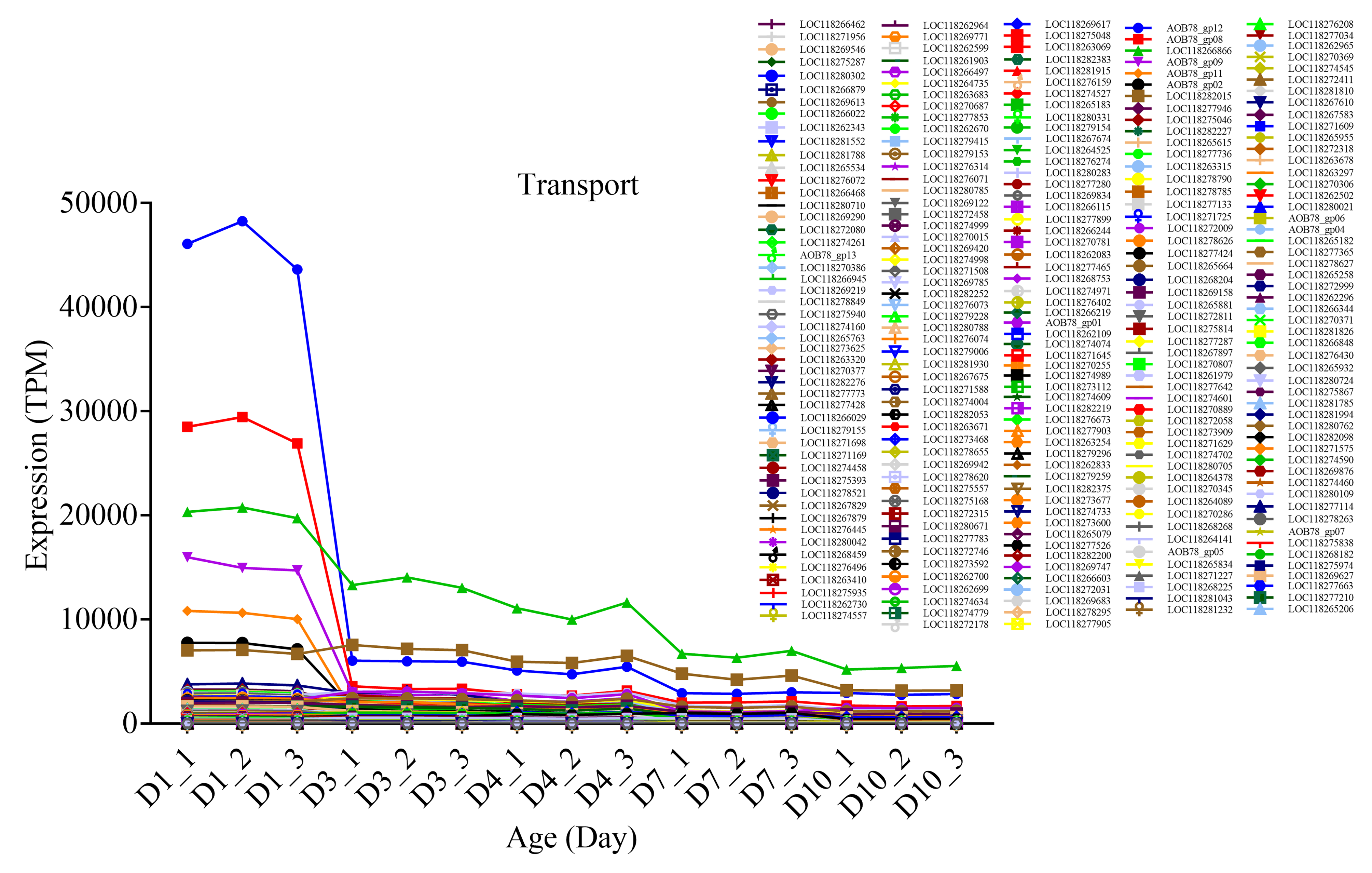

Supplement: Supplementary file 1 [file insects-13-00936-s001.zip › Fig S5.tif]

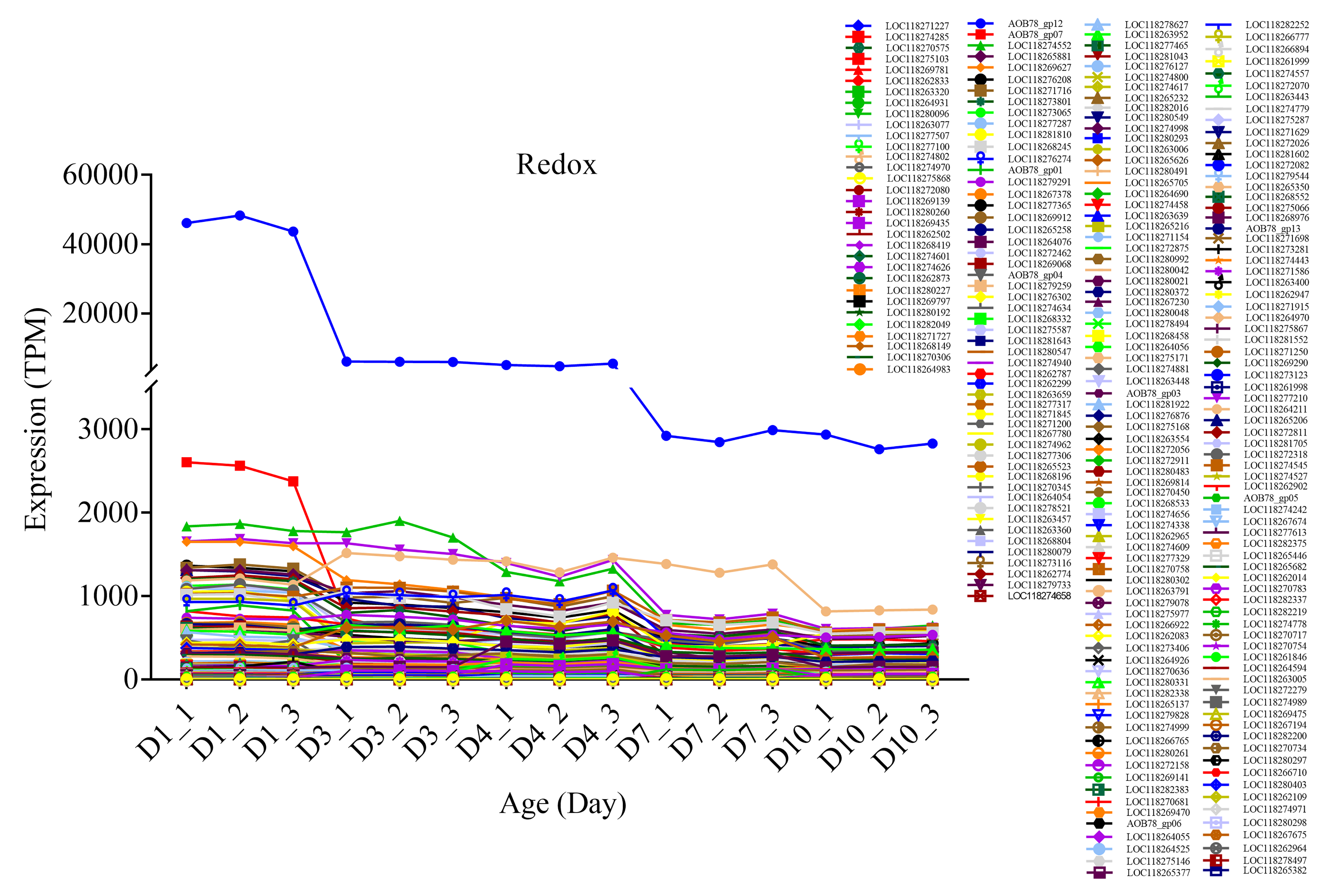

Supplement: Supplementary file 1 [file insects-13-00936-s001.zip › Fig S6.tif]

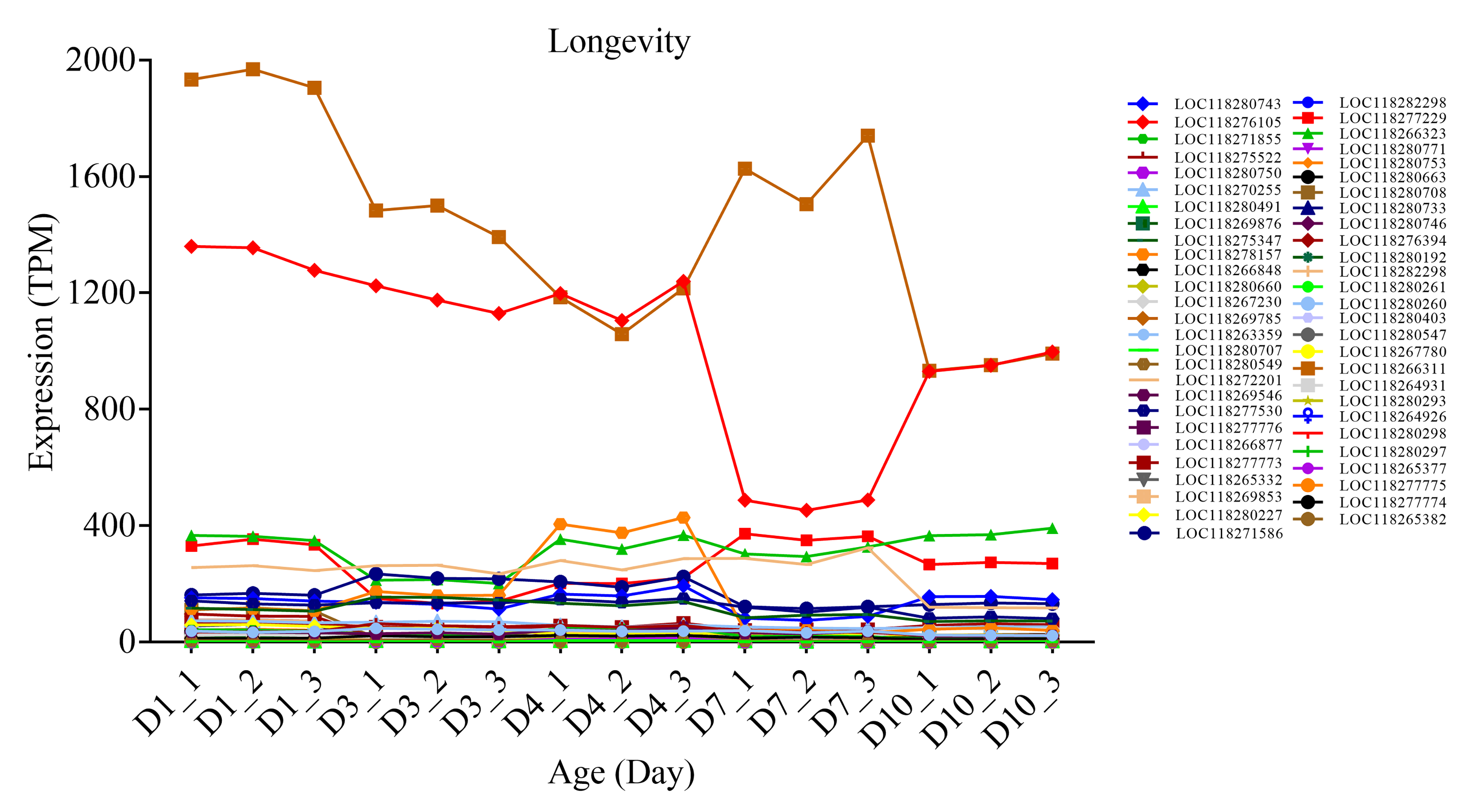

Supplement: Supplementary file 1 [file insects-13-00936-s001.zip › Fig S7.tif]

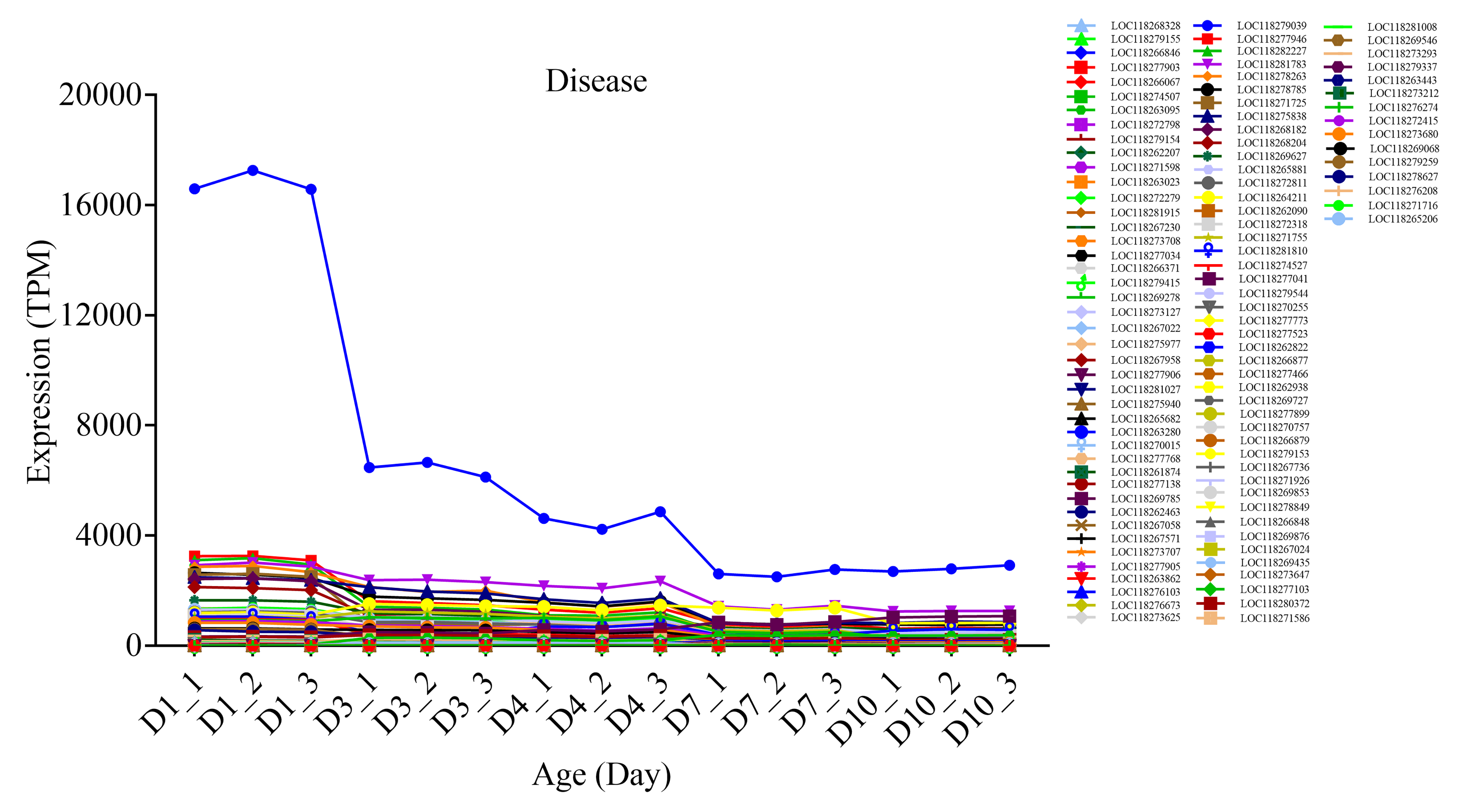

Supplement: Supplementary file 1 [file insects-13-00936-s001.zip › Fig S8.tif]

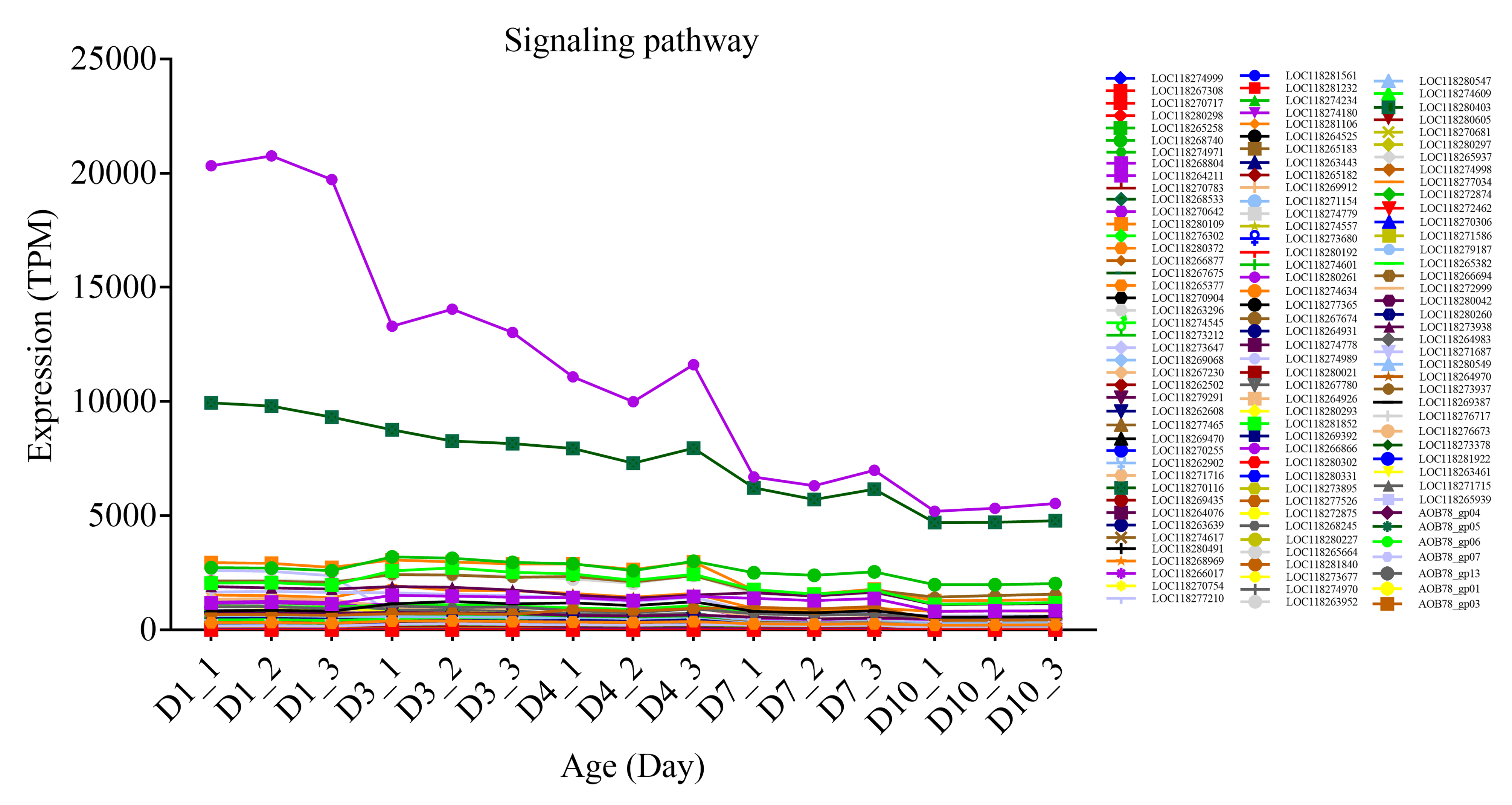

Supplement: Supplementary file 1 [file insects-13-00936-s001.zip › Fig S9.tif]

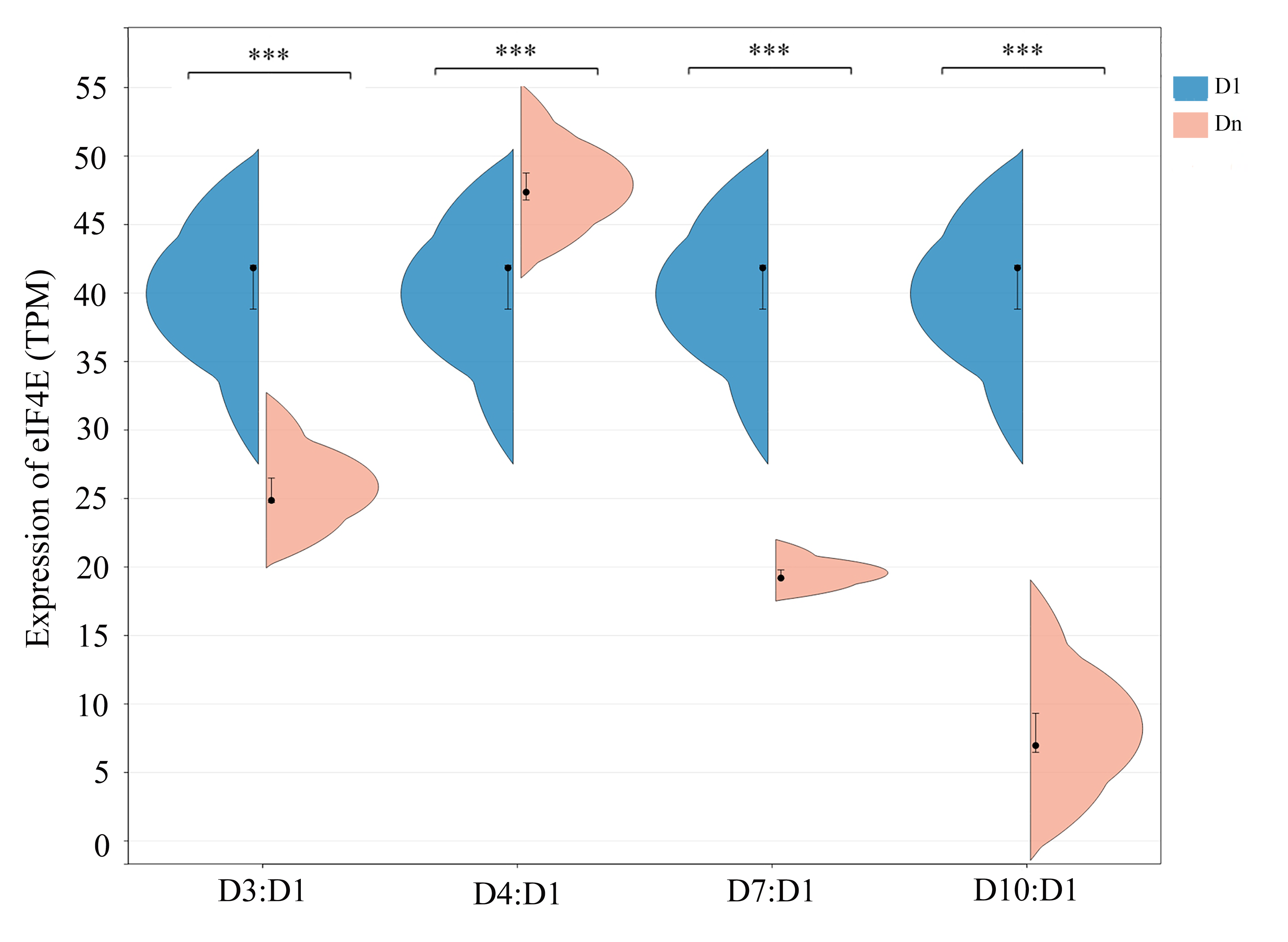

Supplement: Supplementary file 1 [file insects-13-00936-s001.zip › Fig. S16.tif]
